# Comprehensive geriatric assessment predicts mortality and adverse outcomes in hospitalized older adults

**DOI:** 10.1186/1471-2318-14-129

**Published:** 2014-12-03

**Authors:** Thiago J Avelino-Silva, Jose M Farfel, Jose AE Curiati, Jose RG Amaral, Flavia Campora, Wilson Jacob-Filho

**Affiliations:** Geriatrics Division, Internal Medicine Department, University of Sao Paulo Medical School, Sao Paulo, SP Brazil

**Keywords:** Geriatric assessment, Outcomes, Hospital care, Delirium, Nutrition

## Abstract

**Background:**

Comprehensive Geriatric Assessment (CGA) provides detailed information on clinical, functional and cognitive aspects of older patients and is especially useful for assessing frail individuals. Although a large proportion of hospitalized older adults demonstrate a high level of complexity, CGA was not developed specifically for this setting. Our aim was to evaluate the application of a CGA model for the clinical characterization and prognostic prediction of hospitalized older adults.

**Methods:**

This was a prospective observational study including 746 patients aged 60 years and over who were admitted to a geriatric ward of a university hospital between January 2009 and December 2011, in Sao Paulo, Brazil. The proposed CGA was applied to evaluate all patients at admission. The primary outcome was in-hospital death, and the secondary outcomes were delirium, nosocomial infections, functional decline and length of stay. Multivariate binary logistic regression was performed to assess independent factors associated with these outcomes, including socio-demographic, clinical, functional, cognitive, and laboratory variables. Impairment in ten CGA components was particularly investigated: polypharmacy, activities of daily living (ADL) dependency, instrumental activities of daily living (IADL) dependency, depression, dementia, delirium, urinary incontinence, falls, malnutrition, and poor social support.

**Results:**

The studied patients were mostly women (67.4%), and the mean age was 80.5±7.9 years. Multivariate logistic regression analysis revealed the following independent factors associated with in-hospital death: IADL dependency (OR=4.02; CI=1.52-10.58; *p*=*.*005); ADL dependency (OR=2.39; CI=1.25-4.56; *p*=.008); malnutrition (OR=2.80; CI=1.63-4.83; *p*<.001); poor social support (OR=5.42; CI=2.93-11.36; *p*<.001); acute kidney injury (OR=3.05; CI=1.78-5.27; *p*<.001); and the presence of pressure ulcers (OR=2.29; CI=1.04-5.07; *p*=.041). ADL dependency was independently associated with both delirium incidence and nosocomial infections (respectively: OR=3.78; CI=2.30-6.20; *p*<.001 and OR=2.30; CI=1.49-3.49; *p*<.001). The number of impaired CGA components was also found to be associated with in-hospital death (*p*<.001), delirium incidence (*p*<.001) and nosocomial infections (*p*=.005). Additionally, IADL dependency, malnutrition and history of falls predicted longer hospitalizations. There were no significant changes in overall functional status during the hospital stay.

**Conclusions:**

CGA identified patients at higher risk of in-hospital death and adverse outcomes, of which those with functional dependence, malnutrition and poor social support were foremost.

## Background

Hospitalization is frequently required for the treatment of acute or uncontrolled illnesses and for invasive diagnostic procedures in older adults. Nonetheless, hospitalization is also considered a risk event for these patients [1–4]. Elderly individuals suffer physiological changes typical of the aging process that make them more susceptible to adverse events during hospitalization, which may result in a series of complications unrelated to the initial cause of admission. These complications may lead to an increased length of hospital stay, functional decline and higher mortality [1]. Furthermore, one in every three hospitalized older adults loses the ability to perform activities of daily living (ADLs), and at least 20% develop delirium during their hospitalization [2, 5, 6]. Even so, evidence suggests that health care providers have low levels of awareness concerning the risks of hospitalization in this population [7].

The early identification of individuals at greatest risk for complications and unfavorable outcomes would enable a more adequate treatment plan and a better allocation of the resources available to the multidisciplinary team [8]. Moreover, while greater efficiency might be achieved in the proposed treatments, patients and families may be better prepared for the subsequent difficulties that follow hospital discharge. For this purpose, a systematic assessment upon hospital admission may be helpful [8].

The term “Comprehensive Geriatric Assessment” (CGA) was first used in the United Kingdom in the late 1930s. Later, its concept, parameters and indications motivated various scientific research studies [9]. The basic concepts and parameters used in CGAs have evolved over the years, including elements of traditional clinical examinations, evaluations conducted by social workers, functional evaluations performed by rehabilitation specialists, nutritional assessments and neuropsychological evaluation methods [10]. Such assessments are traditionally directed to the planning of interventions but have also been described as useful to determine prognoses and outcomes [9–12].

The present study sought to develop a protocol for conducting a standardized and structured CGA at the time of hospital admission of older patients. We aimed to evaluate the applicability of the proposed model for thoroughly characterizing these patients and analyzed the impact of this strategy on the prediction of mortality and on adverse hospital outcomes.

## Methods

### Study subjects and setting

We conducted a prospective observational study involving patients admitted to a geriatric ward of a 2,200-bed tertiary university hospital in Sao Paulo, Brazil. The unit consists of 18 beds and admits non-surgical, non-orthopedic patients aged 60 years and over for in-hospital care. Patients are referred due to acute illnesses or chronic illness complications. The unit is staffed with a multidisciplinary team that includes geriatricians, nurses, physiotherapists, speech therapists, social workers, psychologists and nutritionists, all of whom meet weekly to discuss inpatient cases. The study was approved by the Ethics Committee for Analysis of Research Projects of the Hospital Clinical Board and conforms to the provisions of the Declaration of Helsinki.

All patients consecutisevely admitted to the ward from January 01, 2009 to December 31, 2011 were considered for study inclusion. Patients admitted exclusively for end-of-life care were excluded from the analysis so as not to bias the determination of prognostic factors.

### Comprehensive geriatric assessment

A protocol detailing the proposal for the geriatric evaluation of these patients was designed. The evaluations were completed within the first 24 hours of admission and at the end of the hospital stay and were performed by geriatrics fellows under the supervision of permanent staff physicians. These professionals had received previous training for proper application of the proposed scales, thus ensuring homogeneous data collection.

Demographic and medical history data were initially evaluated. Socioeconomic appraisal used the ABIPEME Classification [13], which scores patients according to the head of household’s education level and the household number of colored television sets, radio systems, DVD players, washing machines, refrigerators, bathrooms, automobiles, and domestic employees (range, 0–46; 46 = best score). Subjects who scored 17 points or less and lived alone without care from other family members were regarded as having poor social support. Histories of falls and urinary incontinence were assessed with the Debrief of Falls [14] and the Three Incontinence Questions [15], respectively. Polypharmacy was defined as the regular use of 5 or more medications. Acute kidney injury diagnosis followed Acute Kidney Injury Network (AKIN) criteria [16].

Current and previous functional status were measured by ADLs [17–22] and instrumental activities of daily living (IADLs) [19–21, 23–25]. ADLs were scored numerically, with higher numbers representing better functioning (range 0–12; 12 = best score), as were IADLs (range 0–18; 18 = best score) [25]. Previous baseline functionality was defined as the status at 3 months prior to admission. Patients with one or more dependencies in ADLs or IADLs were considered ADL-dependent or IADL-dependent, respectively. Patients with dementia were additionally assessed according to Functional Assessment Staging [26, 27].

Cognitive function was evaluated using the Mini-Mental State Examination (MMSE) [28, 29] and the Informant Questionnaire on Cognitive Decline in the Elderly (IQCODE) [30, 31]. The IQCODE was modified to consider the status at 3 months prior to admission as the baseline condition, thus avoiding distortions due to acute clinical problems. Patients were classified as possibly demented when MMSE, IQCODE and previous functional status were altered. Depression diagnosis was based on the Mini International Neuropsychiatric Interview [32–34], the Geriatric Depression Scale [35, 36], and the Cornell Scale for Depression in Dementia [37–39]. Patients were also evaluated with daily application of the Confusion Assessment Method (CAM) for delirium detection [40, 41]. When positive, patients were further assessed for delirium severity with the Delirium Index [42].

Nutritional evaluation was based on the Mini Nutritional Assessment (MNA) [43, 44]. Malnutrition was defined by a MNA score of 17 or less combined with serum albumin levels lower than 3.5 g/dL. Laboratory tests, selected by the prognostic value defined in previous studies, were also routinely collected within the first 24 hours of hospitalization and included hemoglobin, leukocyte count, creatinine, urea, C-reactive protein, and albumin [6, 45]. Glomerular filtration rate was estimated using the Modification of Diet in Renal Disease Study Group (MDRD) formula [46].

Risk assessment was established using the Charlson Comorbidity Index [47], the Cumulative Illness Rating Scale for Geriatrics (CIRS-G) [48], and the Burden of Illness Score for Elderly Patients (BISEP) [45]. Data related to hospitalization, including new diagnoses, occurrence of delirium and infections were recorded upon hospital discharge or death. The information collected in this study provided a database for future epidemiological, clinical and laboratory studies on predictors of clinical outcomes.

### Outcome variables and CGA components

The primary outcome variable was the occurrence of in-hospital death. In-hospital adverse events, such as delirium, nosocomial infections and functional decline, were also investigated. Factors associated with length of stay were also analyzed; the median days of hospitalization was used as cut-off for classifying length of stay as longer or shorter. Impairment in ten CGA components were particularly investigated for association with these outcomes: polypharmacy; ADL dependency; IADL dependency; depression; dementia; delirium; urinary incontinence; falls; malnutrition; and poor social support.

### Statistical analysis

A descriptive statistical analysis of baseline demographic, clinical and laboratory characteristics, and the outcomes of hospitalization was performed. Categorical variables were compared in each group using contingency tables and tested using the Chi-squared test. Continuous variables were compared using the Student *t* or Mann–Whitney tests, and their correlation was tested using the Pearson or Spearman methods, according to their distribution of normality. Multivariate binary logistic regression was performed to assess independent factors associated with mortality, delirium incidence, nosocomial infections and longer hospital stays. Multivariate analysis included variables that yielded *p* values of 0.1 or lower in the initial univariate analysis. An alpha error of 5% was accepted. In order to assess the possibility of period effects in the results, outcome frequencies were also compared throughout the different semesters of the study. Tests were performed using the IBM statistical software SPSS Statistics for Mac, version 21.0 (Armonk, NY: IBM Corp).

## Results

In total, 746 cases were included in this study from an initial sample of 826 patients; 38 (4.6%) subjects admitted for end-of-life care were excluded; 42 (5.1%) cases were excluded from the analysis because of incomplete assessments. Reasons for inadequate completion of the protocols included the absence of informants accompanying patients with altered cognition (73.8%) and medical staff incompliance (26.2%). Regardless, adherence to the protocol exceeded 95%, and the evaluations took an average of 60 minutes to be performed.

The mean age of the population was 80.7 (±8.1) years, with 65.7% (490) of the participants identified as female and 38.1% (284) as married individuals. The mean years of education were 4.6 (± 3.6), and 37.9% (283) of the patients had low or very low socio-economic levels. At admission, 62.1% (463) of the patients were regularly using 5 or more medications. Further population characteristics and CGA component frequencies are outlined in Tables 1 and 2, respectively.

**Table 1:** **Characteristics of the study population at admission and univariate analysis according to in-hospital death**

	Total (n = 746)	No death (n = 650)	Death (n = 96)	***P***-value
**Demographics**				
**Age**	80.7 ± 8.1	80.5 ± 7.9	81.6 ± 9.3	.173
**Female, n (%)**	490 (65.7)	438 (67.4)	52 (54.2)	.011
**Married, n (%)**	284 (38.1)	246 (37.8)	38 (39.6)	.744
**Comorbidities**				
**Hypertension, n (%)**	682 (78.0)	514 (79.1)	68 (70.8)	.069
**Diabetes, n (%)**	250 (33.5)	222 (34.2)	28 (29.2)	.334
**Heart failure, n (%)**	206 (27.6)	180 (27.7)	26 (27.1)	.901
**Coronary disease, n (%)**	124 (16.6)	106 (16.3)	18 (18.8)	.230
**Previous stroke, n (%)**	124 (16.6)	110 (16.9)	14 (14.6)	.219
**Obesity, n (%)**	82 (11.0)	76 (11.7)	6 (6.3)	.112
**Osteoporosis, n (%)**	120 (16.1)	110 (16.9)	10 (10.4)	.105
**Osteoarthritis, n (%)**	112 (15.0)	102 (15.7)	10 (10.4)	.177
**Cancer, n (%)**	106 (14.2)	92 (14.2)	14 (14.6)	.910
**COPD, n (%)**	76 (10.2)	70 (10.8)	6 (6.3)	.172
**Acute illness and complications**				
**Acute kidney injury, n (%)**	156 (20.9)	118 (18.2)	38 (39.6)	<.001
**Pressure ulcer, n (%)**	50 (6.7)	34 (5.2)	16 (16.7)	<.001
**Weight loss, n (%)**	196 (26.3)	174 (26.8)	22 (22.9)	.423
**Infection, n (%)**	286 (38.3)	236 (36.3)	50 (52.1)	.003
**Risk assessment**				
**CCI**	3.7 ± 2.3	3.7 ± 2.2	3.6 ± 2.2	.433
**BISEP**	3.0 ± 1.5	2.8 ± 1.5	3.7 ± 1.5	<.001
**CIRS-G**	10.3 ± 5.0	9.9 ± 4.7	14.1 ± 6.2	.002
**Laboratory**				
**Hemoglobin (g/dL)**	11.4 ± 2.3	11.4 ± 2.3	11.4 ± 2.0	.704
**Creatinine clearance (mL/min/1.73 m** ^**2**^ **)**	48.5 ± 23.8	48.5 ± 22.9	48.3 ± 29.2	.453
**Albumin (g/dL)**	3.6 ± 0.6	3.6 ± 0.6	3.0 ± 0.6	<.001
**Hospital stay (days)**	16.7 ± 14.5	16.3 ± 14.4	18.9 ± 14.7	.064

COPD = Chronic Obstructive Pulmonary Disease; CCI = Charlson Comorbidity; BISEP = Burden of Illness Score for Elderly Persons; CIRS-G = Cumulative Illness Rating Scale for Geriatrics.

**Table 2:** **Univariate analysis of comprehensive geriatric assessment components according to in-hospital death**

	Total (n = 746)	No death (n = 650)	Death (n = 96)	***P***-value
**Number of medications**	5.4 ± 3.5	5.2 ± 3.2	6.9 ± 3.5	<.001
**ADLs (range 0–12; 12 = best)**	7.3 ± 4.7	7.7 ± 4.5	4.4 ± 2.8	<.001
**ADL dependency, n (%)**	292 (48.0)	252 (46.0)	40 (66.7)	.002
**IADLs (range 0–18; 18 = best)**	7.2 ± 6.2	7.9 ± 7.2	2.8 ± 2.2	<.001
**IADL dependency, n (%)**	409 (67.3)	355 (64.8)	54 (90.0)	<.001
**Depression, n (%)**	224 (30.0)	206 (31.7)	18 (18.8)	.010
**Dementia, n (%)**	194 (26.0)	156 (24.0)	38 (39.6)	.001
**Prevalent delirium, n (%)**	138 (18.5)	102 (15.7)	36 (37.5)	<.001
**Urinary incontinence, n (%)**	350 (46.9)	304 (46.8)	46 (47.9)	.833
Falls*, n (%)	96 (12.9)	84 (12.9)	12 (12.5)	.908
**Malnutrition**	314 (42.1)	242 (37.2)	72 (75.0)	<.001
**Poor social support**	203 (27.2)	141 (21.7)	62 (64.6)	<.001

*Two or more falls during the last 12 months.

ADLs = Activities of Daily Living; IADLs = Instrumental Activities of Daily Living.

**Figure 1:**
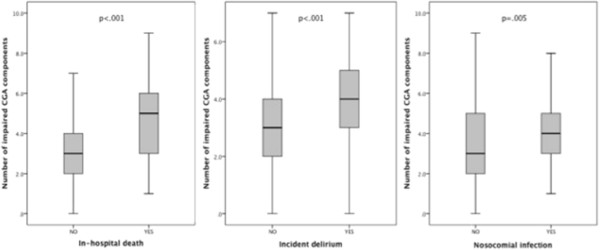
**Number of impaired comprehensive geriatric assessment components according to adverse outcomes.**

The overall mortality rate was 12.9% (96), and the leading cause of death was septic shock (46.7%) followed by cardiovascular complications (19.6%) and neoplastic disease complications (12.5%). Characteristics according to all-cause mortality can be found in Table 1. Compared with the patients who were discharged, those who died had a significantly higher number of impaired CGA components (Table 2; Figure 1), including functional dependency, cognitive decline and polypharmacy. Multivariate binary logistic regression indicated that IADL dependency, ADL dependency, malnutrition, poor social support, acute kidney injury and pressure ulcers at admission were all independently associated with in-hospital death (Table 3). The importance of malnutrition markers stood out, and the average score on the MNA was lower in patients who died (14.3 ± 5.9 *vs.* 18.2 ± 5.4; *p <* .001), with a good correlation between this score and albumin levels at admission (rho = 0.5; *p <* .001). Both the BISEP and CIRS-G scores served as predictors of in-hospital mortality in this population, though with a weak correlation between tests (rho = .14; *p =* .017). We also verified that neither mortality (*p =* .58), nor the frequency of nosocomial infections (*p =* .11), delirium (*p =* .32) or longer hospital stays (*p =* .11) significantly varied during the study extent.

**Table 3:** **Independent predictors of in-hospital death and adverse outcomes, after multivariate logistic regression**

	OR	95% CI	***p***
**In-hospital death**			
**IADL dependency**	4.02	1.52-10.58	.005
**ADL dependency**	2.39	1.25-4.56	.008
**Malnutrition**	2.80	1.63-4.83	<.001
**Poor social support**	5.42	2.93-11.36	<.001
**Acute kidney injury**	3.05	1.78-5.27	<.001
**Presence of pressure ulcer**	2.29	1.04-5.07	.041
**Delirium incidence**			
**IADL dependency**	3.52	1.63-7.62	.001
**ADL dependency**	3.78	2.30-6.20	<.001
**Malnutrition**	1.95	1.35-2.80	<.001
**Dementia**	3.0	2.04-4.40	<.001
**Nosocomial infections**			
**ADL dependency**	2.30	1.49-3.49	<.001
**Longer hospital stay**			
**IADL dependency**	2.40	1.69-3.40	<.001
**Malnutrition**	1.46	1.10-1.98	.016
Falls*	1.81	1.16-2.83	.009

*Two or more falls during the last 12 months.

ADLs = Activities of Daily Living; IADLs = Instrumental Activities of Daily Living.

The number of impairments among the 10 analyzed CGA domains was also significantly associated with the incidence of delirium and nosocomial infections (Figure 1). In total, 88 patients developed delirium while hospitalized, with a 26.1% mortality. These patients were more frequently male (40.9 *vs.* 28.8%; *p <* .001), demented (35.2 *vs.* 15.2%; *p <* .001), ADL and IADL dependent (75% vs. 40%, *p <* .001), malnourished (59.1 *vs.* 34.2%; *p <* .001), and infected at admission (40.9 *vs.* 29.6; *p =* .035). ADL and IADL dependency, malnutrition, and dementia were independently associated with this complication (Table 3). In total, 124 patients had nosocomial infections, of which 48.4% were respiratory infections and 27.4% were urinary tract infections; mortality reached 30.6% in this group. ADL dependency was frequent (72.6 *vs*. 53.7%; *p <* .001) and independently associated with nosocomial infections (OR = .89/95%CI = .85-.93/*p <* .001).

The mean and median lengths of stay were high (16.7 and 12.0 days, respectively), and the following CGA components were related to longer hospitalizations: IADL dependency, malnutrition and history of falls (Table 3). Only 52.0% (454) of the subjects were independent in all ADLs at admission and 32.7% (337) in all IADLs. Notably, 3 months before admission, the mean ADL and IADL scores were 8.9 ± 4.1 and 8.5 ± 7.1, respectively, while at admission, these scores had decreased to 7.3 ± 4.7 and 7.2 ± 6.2, indicating a significant functional decline (*p <* .001). Despite these findings, there were no significant changes in overall functional status during the hospital stay, nor were factors identified that could reliably predict the functional evolution throughout this period.

The mean IQCODE score was 3.8 ± .8, and the mean MMSE score among non-delirious patients was 19.3 ± 8.0. The systematic assessment of cognition associated with functional evaluations enabled the detection of 134 possible new cases of dementia among patients who had not been diagnosed during their outpatient monitoring. Furthermore, screening for prevalent delirium identified 154 (19.6%) cases of the condition. Among these subjects, those who died in the hospital presented a higher mean Delirium Index score at admission (15.5 ± 4.1 vs. 12.2 ± 3.5; *p =* .020).

## Discussion

The importance of CGA emerges in environments such as the geriatric ward, recognizing that not only medical conditions but also social, neuropsychological, nutritional and environmental factors are crucial to the clinical evaluation [9, 10, 49, 50]. In such settings, we find a high frequency of individuals with cognitive impairment, functional dependence and malnutrition, as demonstrated in our results.

Recent studies have also investigated the use of CGA as a prognostic instrument and concluded that several of its components are cornerstones for clinical decision-making [12, 51, 52]. Our model proved valuable precisely in the detection of these key aspects and demonstrated that the functional, cognitive, nutritional and social components of CGA are predictors of in-hospital mortality. Various CGA domains also predicted other adverse outcomes, such as delirium incidence, nosocomial infections and longer hospital stays. Functional dependency was an especially important predictor of these events. Knowing this, early rehabilitation strategies are followed in our unit and possibly explain why no significant changes in overall functionality were observed throughout the hospitalization. Previous studies proposing early rehabilitation interventions have been able to prevent in-hospital functional decline, though not necessarily reverse it, indicating that post-discharge programs are essential to return patients to independence [53, 54]. Likewise, nutritional support and supplementation should be considered. The negative impact of social deprivation on prognosis is well established but poorly understood, and additional work is necessary to understand how to alleviate its effect on morbidity and mortality [55]. Finally, the importance of other indicators of clinical severity should not be forgotten, demonstrated herein by the impact of acute kidney injury on prognosis and by the usefulness of scales that reflect burden of illness, such as the BISEP and CIRS-G [56].

We verified an improvement in the detection of cognitive deficits, particularly the advance in the diagnoses of chronic conditions and the early identification of acute confusional states. That possibly 40% of cases of dementia had gone undetected in the outpatient setting is worrisome and should trigger a revision of follow-up strategies. Regarding the recognition of delirium at admission, previous data in the same setting, prior to the routine application of the CAM, indicated a prevalence of the condition of only 5.2% [6]. After the inclusion of the instrument in our CGA, the number increased almost four-fold. Though not independent predictors of mortality in this population, we confirmed the importance of prevalent delirium and dementia as associated factors to unfavorable outcomes and their accurate recognition is essential to potentially improve the quality of in-hospital care.

A limitation to this study is that we did not collect data to formally recognize frail individuals—a subset of patients for whom CGA can be particularly useful. The high frequency of multi-component CGA abnormalities that was found indicates that this was a high-risk group for the development of geriatric syndromes and that many subjects were likely to be frail individuals. Future studies on the association between in-hospital CGA and frailty characteristics are necessary to better understand the syndrome in the hospital setting.

A drawback that restricts the systematic implementation of CGA is that it is time consuming, as we observed in our results. However, we also found that hospitalization, by allowing more time to assess each patient, provided the possibility for a detailed and structured clinical evaluation. Regarding the results, the elevated presence of totally dependent and cognitively impaired patients associated to a floor effect in the functional measurements that were employed, might have played a part in the lack of functional variability that was described. Also, despite the subjects of this analysis having similar characteristics to those of previously reported studies [8, 57], this was a single-center study and our findings have limited generalizability.

Another limitation is that we did not address the long-term effects of using CGA in hospitalized older adults. Research focusing on its impact on post-discharge mortality, institutionalization and re-admissions should be pursued. Furthermore, controlled studies would be helpful to establish causality relations and to eliminate confusion factors. Homogeneous models of assessment must be further developed for the results to be comparable and for the best assessment strategies to be identified [58–61].

## Conclusion

The systematic incorporation of a standardized and scientifically based method of baseline assessment of hospitalized older patients aims to optimize patients’ clinical and functional outcomes and quality of life by increasing the overall detection of modifiable factors and implementing adequate care. The validity of CGA for identifying factors associated with the occurrence of death and other adverse outcomes in the setting of a geriatric in-patient unit was shown herein, as was the importance of thorough cognitive, functional, social and nutritional evaluations. Such care is critical to elucidate fundamental conditions for the therapeutic decision process.
